# Metabolic consequences of cystinuria

**DOI:** 10.1186/s12882-019-1417-8

**Published:** 2019-06-20

**Authors:** Lauren E. Woodard, Richard C. Welch, Ruth Ann Veach, Thomas M. Beckermann, Feng Sha, Edward J. Weinman, Talat Alp Ikizler, Jay A. Tischfield, Amrik Sahota, Matthew H. Wilson

**Affiliations:** 1 Department of Veterans Affairs, Tennessee Valley Healthcare System, Nashville, TN 37212 USA; 2 Vanderbilt Center for Kidney Disease (VCKD), Nashville, TN 37232 USA; 3 Division of Nephrology and Hypertension, Department of Medicine, Vanderbilt University Medical Center, 1161 21st Ave South, MCN S-3223, Nashville, TN 37232 USA; 4 Department of Biomedical Engineering, Vanderbilt University, Nashville, TN 37232 USA; 5 Center for Stem Cell Biology, Vanderbilt University, Nashville, TN 37232 USA; 6 Department of Veterans Affairs, Michael E. DeBakey VA Medical Center, Houston, TX 77030 USA; 7 Department of Genetics, Rutgers University, Piscataway, NJ 08854 USA; 8 Department of Pharmacology, Vanderbilt University Medical Center, Nashville, TN 37232 USA

**Keywords:** Cystinuria, Cystine, Chronic kidney disease, Kidney stones, Nephrolithiasis

## Abstract

**Background:**

Cystinuria is an inherited disorder of renal amino acid transport that causes recurrent nephrolithiasis and significant morbidity in humans. It has an incidence of 1 in 7000 worldwide making it one of the most common genetic disorders in man. We phenotypically characterized a mouse model of cystinuria type A resultant from knockout of *Slc3a1*.

**Methods:**

Knockout of *Slc3a1* at RNA and protein levels was evaluated using real-time quantitative PCR and immunofluorescence. *Slc3a1* knockout mice were placed on normal or breeder chow diets and evaluated for cystine stone formation over time suing x-ray analysis, and the development of kidney injury by measuring injury biomarkers. Kidney injury was also evaluated via histologic analysis. Amino acid levels were measured in the blood of mice using high performance liquid chromatography. Liver glutathione levels were measured using a luminescent-based assay.

**Results:**

We confirmed knockout of *Slc3a1* at the RNA level, while *Slc7a9* RNA representing the co-transporter was preserved. As expected, we observed bladder stone formation in *Slc3a1*^−/−^ mice. Male *Slc3a1*^−/−^ mice exhibited lower weights compared to *Slc3a1*^+/+^. *Slc3a1*^−/−^ mice on a regular diet demonstrated elevated blood urea nitrogen (BUN) without elevation of serum creatinine. However, placing the knockout animals on a breeder chow diet, containing a higher cystine concentration, resulted in the development of elevation of both BUN and creatinine indicative of more severe chronic kidney disease. Histological examination revealed that these dietary effects resulted in worsened kidney tubular obstruction and interstitial inflammation as well as worsened bladder inflammation. Cystine is a precursor for the antioxidant molecule glutathione, so we evaluated glutathione levels in the livers of *Slc3a1*^−/−^ mice. We found significantly lowered levels of both reduced and total glutathione in the knockout animals.

**Conclusions:**

Our results suggest that that diet can affect the development and progression of chronic kidney disease in an animal model of cystinuria, which may have important implications for patients with this disease. Additionally, reduced glutathione may predispose those with cystinuria to injury caused by oxidative stress.

Word count: 327.

**Electronic supplementary material:**

The online version of this article (10.1186/s12882-019-1417-8) contains supplementary material, which is available to authorized users.

## Background

Cystinuria is one of the most common autosomal recessive genetic disorders with an incidence of 1 in 7000 worldwide [1]. Patients suffer from significant morbidity and few new options have been developed in the past 20 years [2]. Cystinuria is caused by an inherited defect in the transport of cystine and dibasic amino acids (ornithine, lysine, and arginine) in renal tubular cells. Cystine is not soluble in urine so kidney and bladder stones form when the renal tubule fails to reabsorb the amino acid back into the bloodstream. Recurrent cystine kidney stones are associated with pain, frequent urinary tract infections, bleeding, urinary tract obstruction, need for multiple surgical procedures, and kidney failure [2]. Medical treatment to prevent the formation of cystine stones is not very effective and has many unpleasant side effects [3]. Mutations in the gene encoding a renal amino acid transporter (*SLC3A1*, or the rBAT protein) have been identified in most patients with cystinuria establishing the most common cause of the disease termed cystinuria type I [4]. A second gene *SLC7A9,* encoding for b^0,+^type amino acid transporter 1 (b^0,+^AT) accounts for a smaller fraction of cases [5, 6]. The rBAT protein (*Slc3a1* gene product) heterodimerizes with the b^0,+^AT protein (*Slc7a9* gene product) within proximal tubular cells in the nephron, thereby mediating resorption of cystine and dibasic amino acids from the urine [7]. Knowledge of the genetic basis of cystinuria provides opportunities for developing treatments.

Mouse models of cystinuria type I have been generated. Peters et al. identified a missense mutation in *Slc3a1* in a N-ethyl-N-nitrosourea (ENU) mutagenesis screen in C3HeB/FeJ mice [8]. The authors demonstrated reduced kidney weight in homozygous males as compared to wild type males and homozygous females. Additionally, plasma urea levels were elevated in homozygous males from 13 to 20 weeks old as compared to wild type animals. Livrozet et al. identified a spontaneous mutation in *Slc3a1* in 129SvPasCrl mice resulting in cystinuria [9]. Renal function was not significantly impaired in the mutant mice as measured by serum creatinine measurements. However, they did observe increased macrophages and interstitial fibrosis in the mutant mice. Ercolani et al. demonstrated bladder outlet obstruction in male cystinuria mice on a mixed C57Bl/6 and 129/SvJ backround [10]. The genetic strategy for generation of this cystinuria mouse line has been reported [11]; however, we sought to more fully characterize this cystinuria mouse strain.

Recently, it has been debated whether or not kidney stone formation is a pathogenic process contributing to kidney disease [12]. Blockages produced by crystals of various composition have been reported to lead to chronic kidney disease, acute kidney injury, renal colic, or nephrocalcinosis [13]. Crystal deposits may be found in the nephron, renal vasculature, and/or urinary tract [13]. Beyond direct blockage, additional molecular mechanisms have been explored including involvement of the inflammasome and crystal granuloma formation [14, 15]. It has been observed that cystinuric patients develop chronic kidney disease (CKD) even more commonly than usual stone formers [16–18]. Hypertension has been shown to be associated with CKD in cystinuria patients [19]. Cystinuria has been associated with lower renal function and quality of life compared to other kidney stone formers [20]. Nonetheless, little is known about what contributes to the development of CKD in patients with cystinuria other than stone formation. We sought to more fully characterize the cystinuria mouse strain previously reported by Ercolani et al. by evaluating plasma amino acid levels and to determine if this strain develops CKD over time [10]. Additionally, though one previous report found decreased glutathione levels in leukocytes of patients with cystinuria [21], glutathione levels have not been studied in other tissues or in *Slc3a1*^−/−^ animals.

## Methods

### Animals

*Slc3a1*^−/−^ and wild type mice (male and female) were bred and maintained as described previously and according to the Institutional Animal Care and Use Committee of the Nashville Tennessee Valley Healthcare System VA and Vanderbilt University Medical Center [10, 11]. Normal and breeder chow diets were obtained from Lab Diet (St. Louis, MO) with 5L0D corresponding to normal and 5LJ5 to breeder. Mice were housed at the Nashville Tennessee Valley Healthcare System VA. Measurements of weight and length were performed on mice that were bred and maintained on breeder chow. To measure length, mice were anesthetized with isoflurane and measured from the tip of the nose to the tip of the tail. Animals were randomly assigned to experimental groups. At the conclusion of the study, animals were euthanized in accordance with the American Veterinary Medical Association by CO_2_ gas followed by cervical dislocation.

### Real time-PCR

Mouse tissues were removed and immersed in RNAlater (Qiagen, Valancia, CA) for mRNA expression studies. RNA was extracted using a Qiagen RNeasy Kit. Primer pairs were designed using Primer Express software (Applied Biosystems, Foster City, CA) and GAPDH expression was used as an endogenous control. The mRNA expression levels of *Slc3a1* and *Slc7a9* were assessed by quantitative real time PCR using SYBR green and an ABI 7900HT Sequence Detection System (Applied Biosystems). Primers are listed in Table 1.

**Table 1:** Primers for real time PCR

Primer name	Sequence 5′ to 3′
mGAPDH-F	CTCCACTCACGGCAAATTCAA
mGAPDH-R	GATGACAAGCTTCCCATTCTCG
*Slc3a1*-F	CCCGGGAACGCCCATCACTT
*Slc3a1*-R	CCCACTGCATCGGTGACTTGG
*Slc7a9*-F	TGTGGGTGCCATCAGTCTGGC
*Slc7a9*-R	TGGCCATGGGCAGGTTTCTGT

### Western blot, immunostaining, and histology

Whole cell protein extracts were prepared from ~ 30 μg kidney tissue using a kit for simultaneous isolation of RNA and protein (Macherey-Nagel, Bethlehem, PA) according to the manufacturer’s instructions. Proteins (10–15 μg/lane) were resolved on a 4–12% Bis-Tris gel, transferred to nitrocellulose and analyzed by immunoblot using rabbit anti-SLC3A1 (Proteintech, Rosemont, IL; 16,343–1-AP, 1:800) and mouse anti-β actin (Novus, Centennial, CO; NB600–501, 1:10,000) as a loading control with a Licor Odyssey Infrared Imaging System. For immunofluorescent detection of rBAT, kidney samples from 10 to 12 week old mice kept on a regular diet were harvested, stained, and imaged as previously described except that sections were paraffin embedded and sectioned by the Vanderbilt University Medical Center Translational Pathology Shared Resource [22]. The primary rabbit anti-rBAT (16343–1-AP, Proteintech, Chicago, IL) diluted 1:200 was followed by donkey anti-rabbit Alexafluor 594 (Life Technologies, Waltham, MA) diluted 1:500 to detect the rBAT protein in red. Nuclei are stained with 4,6-Diamidino-2-phylindole (DAPI) in blue. For hematoxylin and eosin (H&E) staining of kidney and bladder samples, the bisected kidneys and bladders with stones removed were placed into 4% paraformaldehyde at 4 °C overnight with rocking. The tissues were paraffin-embedded, sectioned, and stained by the Vanderbilt Translational Pathology Shared Resource Core. Images were acquired on an Olympus BX51 microscope.

### X-ray imaging

The image shown is of fourteen-week-old male *Slc3a1*^−/−^ mice. To monitor stone formation over time, mice were taken to the Vanderbilt University Institute of Imaging Science, anesthetized with isoflourane and oxygen until immobile, then placed in a Faxitron 2000 X-ray machine at setting 35 for an exposure time of 4 s.

### Biomarker and amino acid measurements

For BUN and creatinine measurements, blood was collected into a microvette CB 300 Z tube with clot activator (Sarstedt, Newton, NC) by submandibular bleed under isoflurane anesthesia with a 5.5 mm lancet. Samples were clotted at room temperature for > 30 min and centrifuged at 4500 rpm for 10–20 min. Serum was immediately aliquoted and stored at − 80 °C for measurement of the blood urea nitrogen by the Vanderbilt University Medical Center Comparative Pathology Laboratory and measurement of creatinine by the University of Alabama-Birmingham O’Brien Center Core C Biomarkers Laboratory by LC-MS/MS. Plasma amino acid concentrations were determined by reverse phase HPLC using a modified version of the methods of Bidlingmeyer et al. [23].

### Glutathione measurements

Glutathione levels were measured using a GSH-GLO Glutathione Assay (Promega, Madison, WI) according to the manufacturer’s instructions. The protein concentration of liver lysates was determined by BCA and concentrations normalized to 400 ng/μl. Liver lysates were assayed +/− 500 μM TCEP. TCEP reduces any oxidized glutathione present in the sample. Luminescence was measured using a FLUOStar-Omega microplate reader.

## Results

**Fig. 1:**
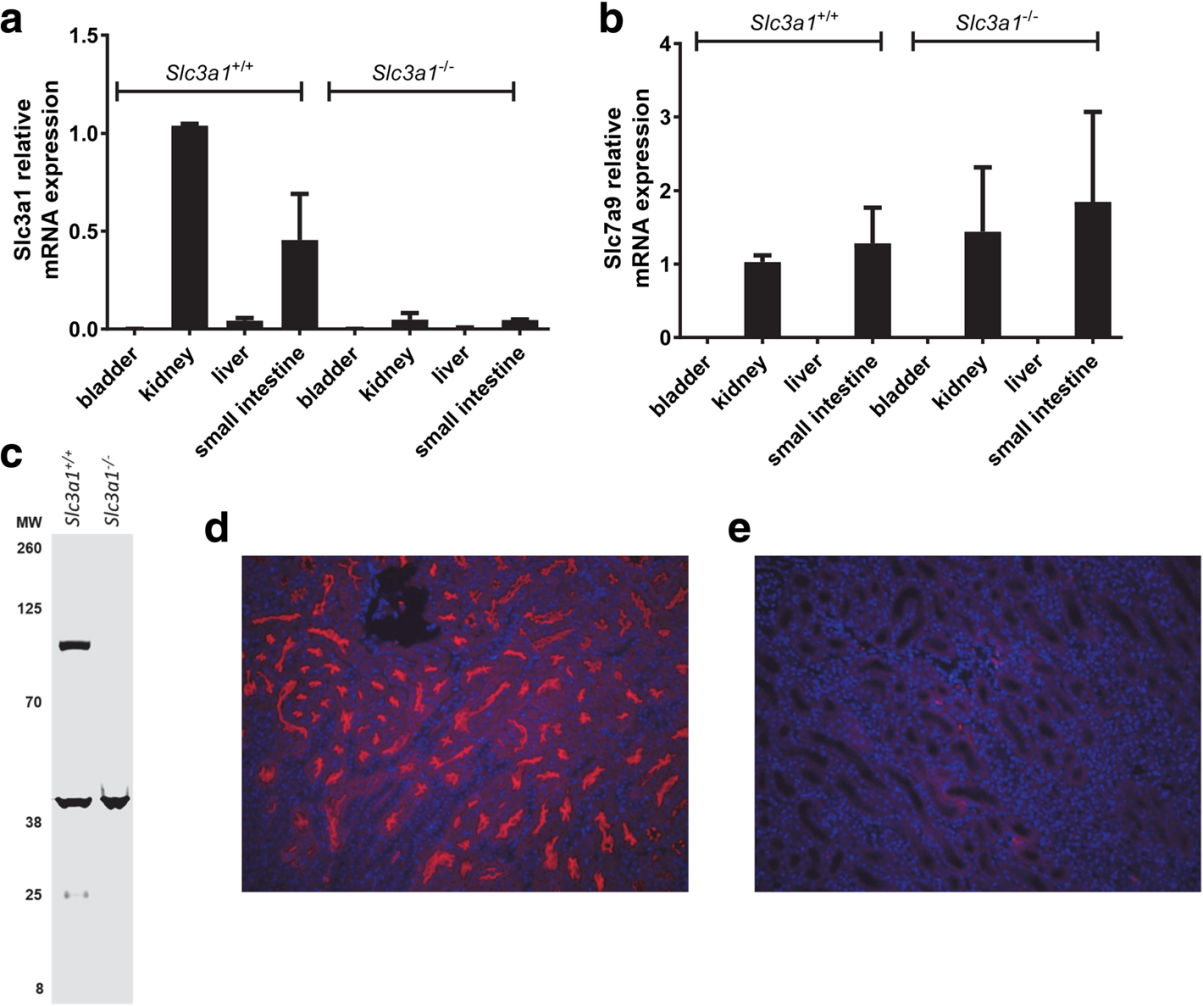
*Slc3a1* transcripts and rBAT protein expression are lost in male *Slc3a1* knockout mice. *Slc3a1*^−/−^ mice demonstrate loss of *Slc3a1* RNA (**a**) but retention of *Slc7a9* RNA (**b**) in kidney and small intestine. Bladder and liver are provided as negative controls. Shown are the averages of two independent experiments done in triplicate (mean ± SD). (**c**) Representative Western blot of rBAT from wild type and *Slc3a1*^−/−^ mice. Immunofluorescence was used to evaluate for rBAT expression in wild type (**d**) and male *Slc3a1*^−/−^ mice (**e**). Knockout animals demonstrate loss of rBAT expression in the proximal tubule

We first confirmed knockout of *Slc3a1* using RT-PCR to evaluate expression levels in various tissues. As expected, *Slc3a1* RNA levels were dramatically reduced in tissues where expression normally occurs, such as the kidney and small intestine (Fig. 1a). Additionally, as expected, *Slc7a9* RNA levels were unaltered by knockout of *Slc3a1* (Fig. 1b)*.* We next used Western blot and immunofluorescent microscopy to evaluate for loss of rBAT expression in kidneys of *Slc3a1*^−/−^ mice. Western blot demonstrated loss of rBAT expression from kidney lysates (Fig. 1c). Immunofluorescent microscopy for rBAT demonstrated that knockout of *Slc3a1* resulted in loss of rBAT expression in the proximal tubules of *Slc3a1*^−/−^ mice when compared the wild type mice (Fig. 1d and e).

**Fig. 2:**
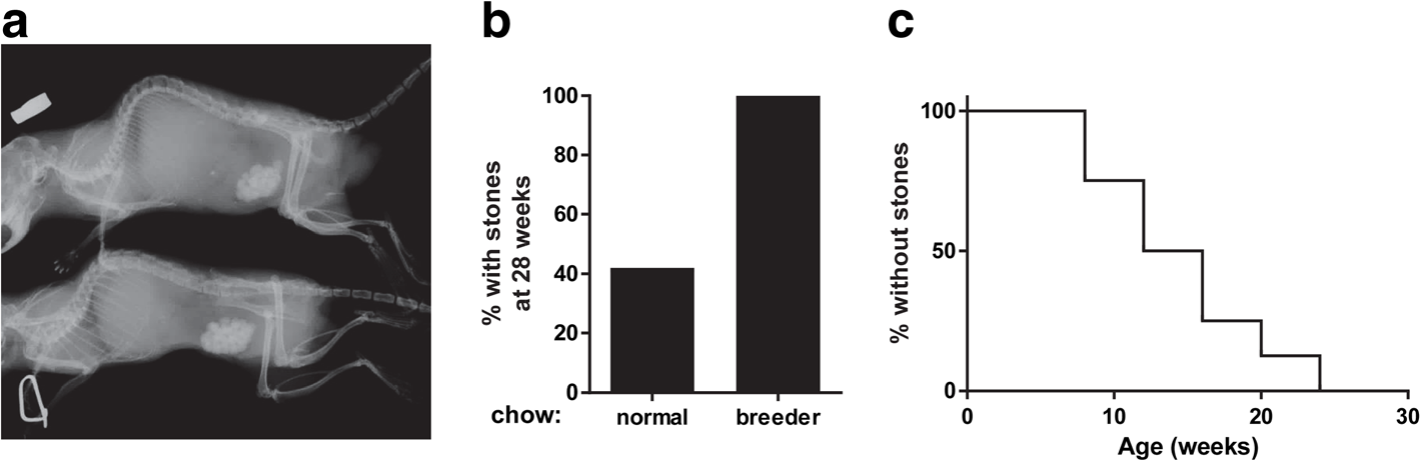
Male *Slc3a1*^−/−^ mice demonstrate differing rates of bladder stone formation dependent upon diet. **a** X-rays of mice with typical bladder stone formation. **b** 42% of mice on normal chow had bladder stones by 28 weeks, whereas 100% of mice had bladder stones by 28 weeks on breeder chow (*N* = 12). **c** X-ray of *Slc3a1*^−/−^ animals revealed the development bladder stone formation on breeder chow as depicted by a Kaplan-Meier plot (*N* = 8)

Knockout mice did not develop kidney stones but did develop bladder stones detectable by x-ray as reported previously (Fig. 2a) [10]. Consistent with previous observations, we observed gender differences between male and female *Slc3a1*^*−/−*^ mice with female mice very rarely developing bladder stones [10]. We did not observe a high rate of bladder stone formation in male *Slc3a1*^−/−^ mice on normal chow (0.31% cystine) (Table 2). However, when we placed the male *Slc3a1*^−/−^ mice on breeder chow (0.36% cystine), we observed a higher rate of bladder stone formation. Comparing the % of mice with bladder stones at 28 weeks, 100% of *Slc3a1*^−/−^ mice on breeder chow had bladder stones whereas only 42% of *Slc3a1*^−/−^ mice on regular chow exhibited bladder stone formation (Fig. 2b). The rate of bladder stone formation in mice on the breeder chow is depicted in Fig. 2c. These results demonstrate that dietary intake can have a major impact on the rate of stone formation in *Slc3a1*^−/−^ mice.

**Table 2:** Comparison of normal and breeder chow diets

	Standard (5L0D)	Breeder (5LJ5)
Crude protein not less than	23%	17%
Crude fat not less than	4.5%	11%
Cystine	0.31%	0.36%
Sodium	0.40%	0.43%

**Fig. 3:**
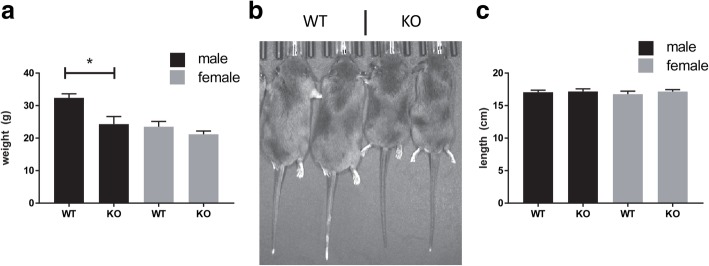
Male *Slc3a1* −/− mice exhibited lower weight compared to wild type mice of the same age. **a** Comparison of weight of 10–12 week old wild type (WT) and *Slc3a1*^−/−^ (KO) animals on breeder chow (*, *p* < 0.05 Mann Whitney test, *N* = 5–6 ± SD). **b** Picture of age matched (12 week old) WT (left) and KO (right) male mice. **c** All mice exhibited the same nose-to-tail length (*N* = 5–6 ± SD)

We observed that male *Slc3a1*^−/−^ mice were consistently lower in weight when compared to *Slc3a1*^+/+^ mice of the same age (Fig. 3a,b). This weight difference was not recapitulated in the female mice. All mice exhibited the same length from their nose to the tip of their tail, indicating an overall metabolic phenotype rather than nutritional deficiency contributed to the difference in weights of the male mice raised and maintained on breeder chow (Fig. 3c). We evaluated the plasma amino acid levels in *Slc3a1*^−/−^ mice and compared them to wild type mice (Table 3). Consistent with the reduced weight in male *Slc3a1*^−/−^ mice, we observed a more severe phenotype with multiple amino acid levels different between wild type and knockout animals. Evaluation of amino acids in both male and female *Slc3a1*^−/−^ mice revealed only ornithine, lysine, and taurine to be reduced in both sexes. Therefore, knockout of rBAT resulted in reduce plasma ornithine and lysine in these animals. Cysteine, which forms cystine when two molecules are joined together, is metabolized to taurine [24] which was also reduced across sexes. Interestingly, plasma cystine was reduced only in male *Slc3a1*^−/−^ despite both male and female mice exhibiting cystinuria [11].

**Table 3:** Plasma amino acid levels (μmole/liter)

	Female		*p*	Male		*p*	Both
	*Slc3a1* ^+/+^	*Slc3a1* ^−/−^		*Slc3a1* ^+/+^	*Slc3a1* ^−/−^		*p*
Aspartate	8.4 ± 1.6	8.5 ± 2.9	0.45	9.44 ± 2.94	9.6 ± 2.1	0.45	
Glutamate	23 ± 2.6	35 ± 19	0.09	50 ± 10.6	46 ± 8.7	0.3	
Serine/Asparagine	61 ± 9.3	46 ± 10	0.012,*	46.1 ± 5.23	51 ± 9.7	0.12	
Glycine	126 ± 17	115 ± 22	0.19	104 ± 11.1	127 ± 15	0.007,*	
Histidine/Glutamine	383 ± 26	363 ± 40	0.16	310 ± 44	416 ± 65	0.004,*	
3-Methyhistidine	258 ± 78	275 ± 66	0.35	57 ± 9.97	46 ± 9.2	0.03,*	
1-Methyhistidine	10.6 ± 1.5	11 ± 3.3	0.29	6.5 ± 0.18	5.9 ± 0.11	3.2e^−5^,*	
Taurine	157 ± 41	101 ± 20	.007,*	108 ± 8.38	92 ± 18	0.04,*	*
Arginine	34 ± 6.9	32 ± 7.4	0.4	27 ± 4.15	30 ± 5.2	0.15	
Threonine/Citruline	240 ± 25	196 ± 38	0.022,*	179 ± 38	149 ± 34	0.09	
Alanine	271 ± 37	224 ± 80	0.11	248 ± 47	196 ± 40	0.03,*	
Proline	76 ± 18	54 ± 15	0.027,*	57 ± 15	45 ± 14	0.1	
Hydroxyproline	10 ± 2.4	6.1 ± 2	0.006,*	8.18 ± 0.88	10 ± 1.2	0.006,*	
Cystine	5.9 ± 3.6	6.2 ± 3.6	0.45	13 ± 3.4	7.05 ± 1.5	0.0008,*	
Tyrosine	45 ± 12	51 ± 13	0.21	37 ± 9.3	34 ± 8.7	0.28	
Valine	129 ± 21	117 ± 27	0.2	110 ± 25	88 ± 24	0.07	
Methionine	69 ± 15	53 ± 17	0.06	57 ± 19.3	46.5 ± 16	0.15	
Ornithine	35 ± 6	11 ± 2	3.7e^−6^,*	34 ± 10	10.5 ± 3.2	0.0001,*	*
Lysine	200 ± 30	86 ± 15	4.5e^−6^,*	147 ± 31	77.4 ± 16	0.0003,*	*
Isoleucine	60 ± 11	59 ± 19	0.45	47 ± 11.6	35.3 ± 11	0.04,*	
Leucine	84 ± 14	84 ± 26	0.48	69 ± 16	54.9 ± 16	0.07	
Phenylalanine	36 ± 5	35 ± 5	0.32	29 ± 4	25.5 ± 4.4	0.047,*	
Tryptophan	34 ± 7	26 ± 8	0.06	30 ± 3	28.1 ± 3.6	0.11	

Plasma amino acid levels were determined as described in the Materials and Methods section. Rows where two amino acids are described (i.e. Histidine/Glutamine) indicates that these amino acids are not separable on the chromatogram. *, indicates a *p* < 0.05 via Student’s T test comparing KO to WT of that sex. The p for “both” indicates that this particular amino acids was reduced in both sexes comparing KO to WT

**Fig. 4:**
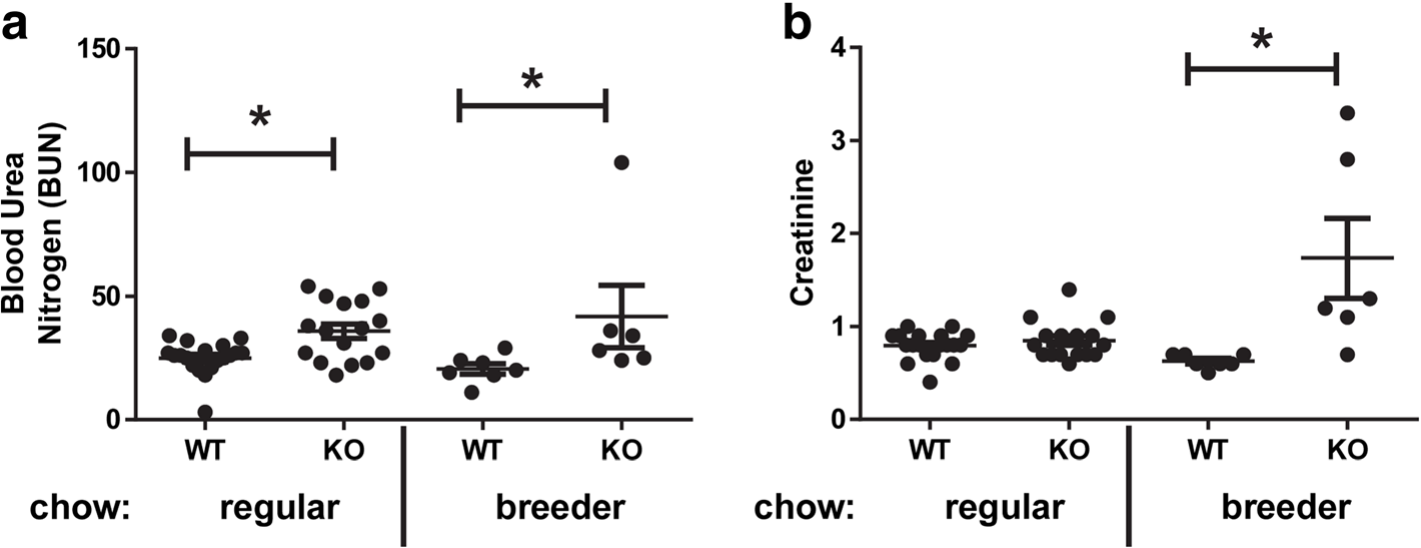
Male *Slc3a1* −/− mice have higher blood urea nitrogen (BUN) and creatinine indicative of the development of CKD. **a** Comparison of BUN in wild type (WT) and *Slc3a1*^−/−^ (KO) animals on a regular or breeder chow diet. KO animals exhibited elevated BUN on both regular and breeder chow when compared to WT animals on the same diet (*, *p* < 0.05 Mann Whitney test, *N* = 6–19 ± SEM). **b** Comparison of serum creatinine in WT and KO animals on a regular or breeder chow diet. KO animals exhibited elevated creatinine on the breeder chow diet but not the regular chow diet (*, *p* < 0.05, Mann Whitney test, *N* = 6–19 ± SEM). Each dot on the graph represents an individual measurement from an individual mouse

**Fig. 5:**
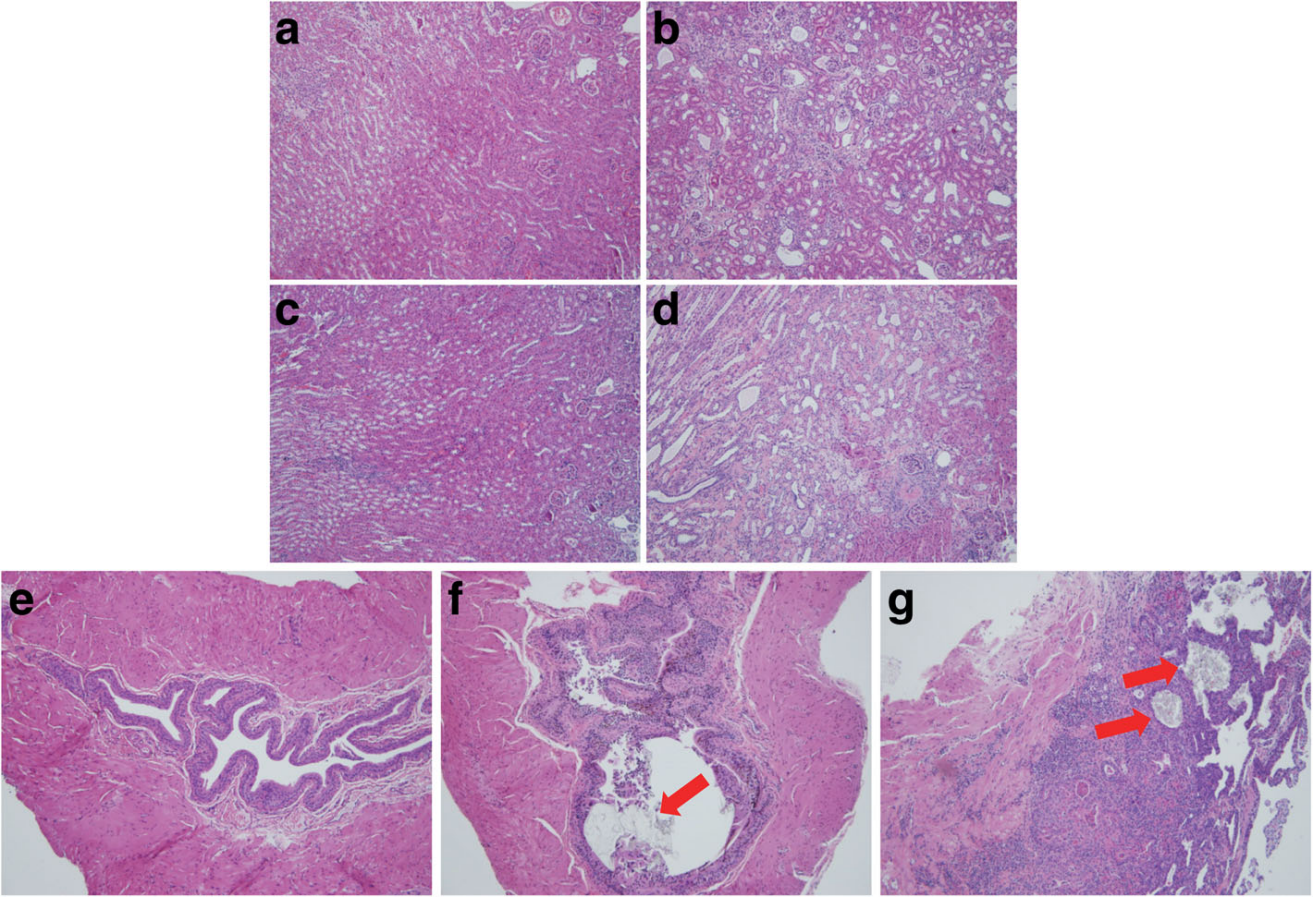
Histological analysis of wild-type and male *Slc3a1*^−/−^ kidneys and bladders at 10–12 months of age. Kidneys of mice with elevated creatinine (**b** and **d**) demonstrate more tubular dilatation and fibrosis when compared to mice with a normal creatinine (**a** and **c**) whether on regular (**a** and **b**) or breeder chow (**c** and **d**). Bladders of wild-type (**e**), knockout with normal creatinine (**f**), and knockout with elevated creatinine (**g**) mice are also shown. The knockouts demonstrate inflammatory infiltrate and cystine crystals. Panel (**g**) demonstrates cystine crystals embedded within the bladder wall (arrows). Shown are representative H&E stains from each group of mice

**Fig. 6:**
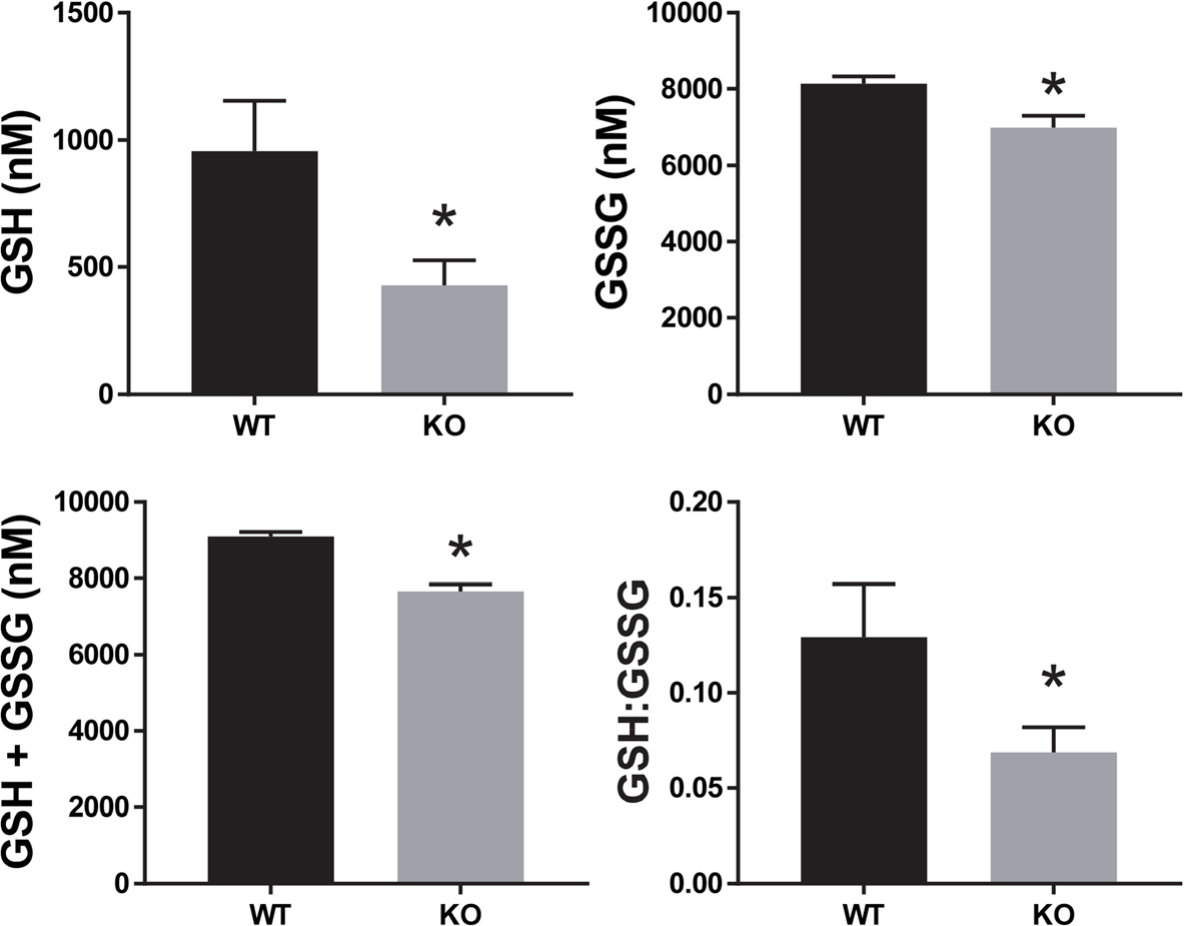
Glutathione levels in male *Slc3a1*^−/−^ mice at 8 weeks of age. Reduced (GSH) and oxidized (GSSG) glutathione levels were measured in the livers of wild-type and *Slc3a1*^−/−^ animals. GSH, GSSG, total and the ratio of reduced to oxidized forms (GSH:GSSG) were all lowered in *Slc3a1*^−/−^ mice (*N* = 6 ± SEM)

Previous reports showed elevation of BUN in some *Slc3a1*^−/−^ mice strains but no elevation in serum creatinine in others [8, 9]. We therefore sought to evaluate for the development of CKD in this mouse strain with a mixed C57Bl/6 and 129/SvJ background [10]. Interestingly, male mice fed only normal chow showed elevated BUN, whereas male mice fed breeder chow showed both elevated BUN and creatinine (Fig. 4). Mice evaluated were between 40 and 87 weeks old (Additional files 1 and 2). Histologic evaluation of the kidney tissue from *Slc3a1*^−/−^ mice with higher creatinine on normal chow (Fig. 5b) demonstrated dilated tubules from obstruction and increased fibrosis when compared to those with normal creatinine (Fig. 5a). The observed dilation of tubules and fibrosis was also observed in mice with elevated creatinine (Fig. 5d) compared to normal creatinine when placed on the breeder chow (Fig. 5c). Histologic evaluation of the bladders of male *Slc3a1*^−/−^ mice revealed increased inflammatory infiltrate within the bladder wall and cystine stones in the bladder in mice having a normal serum creatinine (Fig. 5f) when compared to the bladders of wild type mice (Fig. 5e). Some *Slc3a1*^−/−^ mice with an elevated serum creatinine showed embedded cystine crystals within the bladder wall in addition to bladder inflammatory infiltrate (Fig. 5g).It is known that cystinuria results in stone formation leading to obstruction and CKD. However, we sought to determine if there might be other metabolic consequences of cystinuria beyond just stone formation. Glutathione, or γ-l-glutamyl-l-cysteinyl-glycine (GSH), serves as a detoxifier, protector from oxidative damage, and is arguably the most important low molecular weight antioxidant in cells [25]. The availability of cysteine, formed by two molecules of cystine, is a major determinant of the regulation of GSH synthesis [26]. Therefore, cystine deficiency could affect GSH levels. Martensson et al. previously reported decreased leukocyte GSH levels in homozygous cystinuric patients [21]. We sought to evaluate GSH levels in male *Slc3a1*^−/−^ mice which exhibited lower plasma cystine levels. Therefore, we evaluated the levels of reduced (GSH) and oxidized (GSSG) glutathione in the livers of *Slc3a1*^−/−^ mice. We observed lower GSH and lower GSSG in the livers of *Slc3a1*^−/−^ mice compared to WT controls (Fig. 6). The total amount of glutathione (GSH + GSSG) was lower (Fig. 6), implying a possible connection between the lower availability of cysteine and total glutathione reserve. A reduction of the ratio of reduced to oxidized forms of glutathione was also observed (Fig. 6), suggesting that the ability of the liver to respond to oxidative stress is reduced in *Slc3a1*^−/−^ mice.

## Discussion

Treatment for cystinuria has not dramatically changed in the past 20 years despite greater understanding of the genetic basis [1]. Mouse models for cystinuria not only confirm the genetic basis of the disease but also provide models for testing and evaluating new therapies [10]. There are likely genes that affect stone formation in patients with cystinuria that could be derived from analysis of patients and then tested in mouse models. There are likely genes that affect stone formation in patients with cystinuria that could be derived from analysis of patients and then tested in mouse models. Recently, Zee et al. demonstrated that α-lipoic acid prevents cystine stone formation in the mouse model reported in this article [11].

Three type A cystinuria mouse models have been generated including the one described herein that was generated by exon 1 deletion [10]. The other models consist of spontaneous mutation (E383K) [9] or ENU induced mutagenesis (D140G) [8]. The D140G model on a 129S2/SvPasCrl background demonstrated a 40% reduction in survival by 15 weeks with elevation in serum BUN though no increase in serum creatinine or interstitial kidney fibrosis [9]. Possible gender differences in these observations were not commented on though stones mainly occurred in males [9]. The E383K model on the C3HeB/FeJ background showed elevated serum BUN in 20 week old male mice and decreased kidney weight in 32–48 week old male mice with neither phenotype observed in female mice [8]. The observed gender difference in stone formation between male and female in the context of cystinuria has been observed in patients and reviewed elsewhere [27, 28]. The difference in stone formation does not appear to be due to differences in urinary cystine levels, but it may due to differential cystine aggregation in male versus female urine [27, 29]. Nonetheless, our study demonstrates that CKD does occur in aged male *Slc3a1*^*−/−*^ mice.

Little is known about what factors affect the phenotype of cystinuria and progression to CKD. [2] We found that the diet fed to the *Slc3a1*^−/−^ mice had a dramatic effect on the rate of stone formation. Common treatment for cystinuria includes limiting dietary sodium and protein intake, increased fluid intake, urinary alkalization, and possibly even thiol drugs and captopril [1]. A previous study demonstrated that a low protein diet with more plant protein sources reduced cystine excretion in cystinuria patients [30]. We have not found any studies on the role of diet in cystine stone formation in mouse models of cystinuria. Human clinical studies on the effects of diet for potential modification of human cystinuria are lacking [2]. Future studies could be directed at evaluating dietary changes in a more controlled manner to tease out modifications which could slow the progression of stone formation in cystinuria.

The inability to reclaim cystine from the urine likely alters cystine metabolism in the whole animal. Knockout of *Slc3a1* affects its expression not only in the kidney but also the intestine. The intestinal peptide transporter Pept1 (*Slc15a1*) is thought to permit reabsorption of cystine and other amino acids from the gut somewhat compensating for loss of *Slc3a1* in the intestine [31, 32]. How this might contribute to overall gender difference in cystinuria and our observed differences in plasma amino acid levels is currently unknown.

Our results reveal the metabolic phenotype to be more severe in male knockout animals when compared to females. Glutathione levels were lower in the *Slc3a1*^−/−^ mice. Given the importance of glutathione in detoxification and oxidative stress, cystinuria may result in an overall metabolic state of increased sensitivity to injury from a variety of mechanisms [25]. As patients with cystinuria have been reported to develop more CKD than other stone formers, lower levels of GSH could increase the sensitivity of *Slc3a1*^−/−^ mice to development of and recovery from injury resulting in worsened CKD. Previous research has demonstrated that anti-oxidant cystine metabolites, GSH, and taurine exert protection against progression of renal fibrosis [33–35]. Additionally, others have found these to be protective against renal ischemia reperfusion injury [36–39]. In patients, cystinuria results in kidney stones and obstruction. Reduced glutathione could result in worsened injury or inability to recover from injury in cystinuria when compared to stone formers with non-cystinuric disease pathology. Importantly, new therapies such as α-lipoic acid may reduce stone formation by increasing solubility of cystine in the urine but have no effect on regaining cystine transport [11]. Therefore, such therapy may reduce stone formation but may not improve cystine or oxidative metabolism overall. Our data suggest that diet manipulation, supplementation, and/or drugs that work to improve the ratio of reduced to oxidized glutathione in vivo should be tested in mouse models for their effect on the development of CKD. Such studies would provide further guidance for care of cystinuric patients and hopefully guide the development of new therapies to help prevent kidney disease progression in this population.

## Conclusions

We have demonstrated that dietary intake can have an impact on the severity of the phenotype of cystinuria both in regards to stone formation and the development of CKD in a mouse model of cystinuria. Male mice exhibited a more severe phenotype concerning animal weight and plasma amino acid composition. Cystinuria affected glutathione composition in the liver. Future studies will systematically evaluate dietary components on the cystinuria phenotype and what effect the resultant alteration in glutathione metabolism has on physiology and pathophysiology.

## Additional files

Additional file 1: Supplementary figure demonstrating BUN and creatinine over time in WT and KO mice on regular and breeder chow. (PDF 135 kb)

Additional file 2: Table of WT and KO mice on regular and breeder chow with BUN, creatinine, gross morphology and stone weight. (XLSX 11 kb)

## Data Availability

The datasets used and/or analyzed during the current study are available from the corresponding author on reasonable request.

## Abbreviations

b^0,+^AT: *Slc7a9* gene product
BUN: Blood urea nitrogen
CKD: Chronic kidney disease
DAPI: 4,6-Diamidino-2-phylindole
ENU: N-ethyl-N-nitrosourea
GSH: γ-l-glutamyl-l-cysteinyl-glycine, glutathione
GSSG: Oxidized glutathione
KO: Knockout
rBAT: *Slc3a1* gene product
SEM: Standard error of the mean
WT: Wild type
