# Supplementary material for: Metabolic consequences of cystinuria
Source: BMC Nephrol. 2019 Jun 20;20:227. doi: 10.1186/s12882-019-1417-8 (PMC6585015; doi:10.1186/s12882-019-1417-8)
Supplement: Supplementary file 1 — Supplementary figure demonstrating BUN and creatinine over time in WT and KO mice on regular and breeder chow. (PDF 135 kb) [file 12882_2019_1417_MOESM1_ESM.pdf]

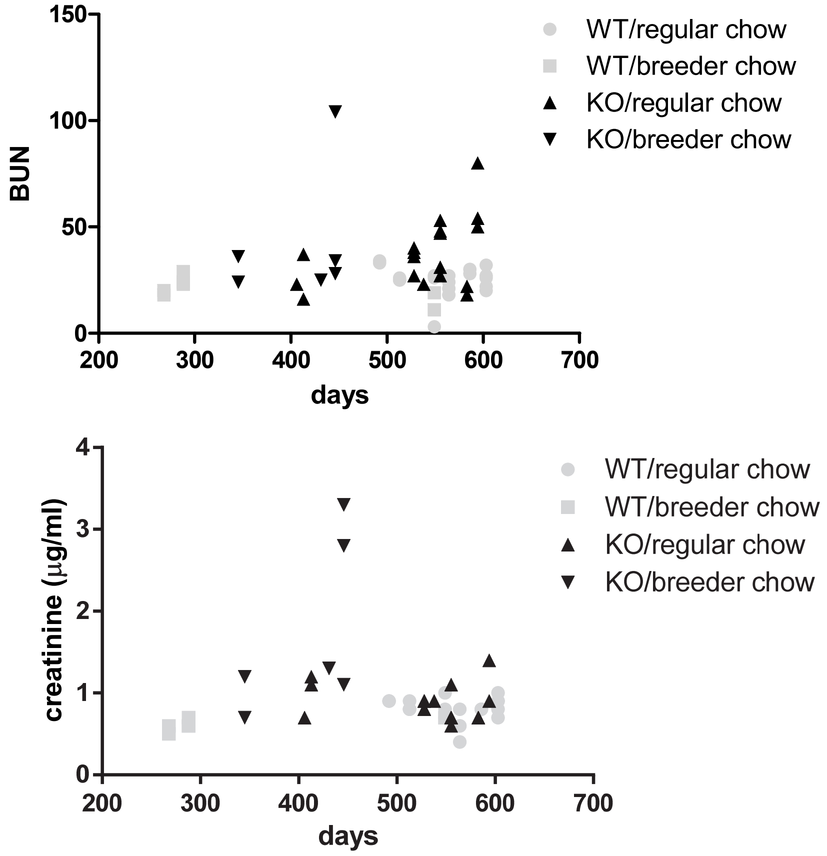

Additional file figure legend: Evaluation of serum BUN and creatinine in male WT or *Slc3a1*$^{-/-}$ mice on regular or breeder chow at a variety of ages. Each point represents a measurement from an individual mouse at that age.

Woodard et al., Additional File 1
